# Controlling refractive index of transformation-optics devices via optical path rescaling

**DOI:** 10.1038/s41598-019-54516-0

**Published:** 2019-12-05

**Authors:** Hossein Eskandari, Tomáš Tyc

**Affiliations:** 1 Department of Electrical Engineering, Ferdowsi University of Mashhad, Mashhad, Iran; 2 Department of Theoretical Physics and Astrophysics, Masaryk University, Kotlářská 2, 61137 Brno, Czechia

**Keywords:** Metamaterials, Transformation optics

## Abstract

We present a general method of designing optical devices based on optical conformal mapping and rescaling the optical path along a given bunch of rays. It provides devices with the same functionality as those based purely on conformal mapping, but enables to manipulate the refractive index to a great extent—for instance, eliminate superluminal regions of space as well as reduce the refractive index in other regions significantly. The method is illustrated in two examples, a waveguide coupler and a plasmonic bump cloak, and numerical simulations confirm its functionality.

## Introduction

Transformation optics (TO) proposed concurrently by Pendry and Leonhardt^1,2^ opened a gateway to put forward ideas for designing devices with unprecedented functionalities. Although the method was used initially to design an invisibility cloak to create a concealment area for hiding an object, researchers soon began to adapt it for various applications like carpet cloaking (also known as plasmonic bump cloaking)^3–8^, polarization splitting and transforming^9–13^, directivity enhancement^14–17^, beam expanding^18,19^, and waveguide coupling^20–24^.

Despite showing promising degrees of freedom to alter the electromagnetic wave propagation, the material derived by this method is in general anisotropic and non-homogeneous and may require extreme values of permittivity and permeability. These properties essentially limit the application of TO for practical purposes.

To simplify the material, a new branch of transformations was proposed. Conformal and quasi-conformal transformations solve the issue of anisotropy and magnetic medium requirements and eventually result in a non-homogeneous dielectric transformation medium^3–5,25–29^. Such dielectric graded index medium can be fabricated by various methods including drilling sub-wavelength holes in a dielectric substrate^5,30,31^, dielectric layered shell deposition^32–34^, parallel waveguide plates height modification for mode index alteration^35–37^, graded photonic crystals^15,38^, electron-beam lithography^39–42^ and modification of the guiding silicon substrate height^41,43^.

Conformal TO has been used for designing various electromagnetic elements like unidirectional cloak^2^, waveguide bends^27,28^ and directivity enhancers^14,15,28^. Recently conformal transformation has been employed to compress the common circular generalized Maxwell fish-eye lens to an elliptical one^44^. However, in some cases, a conformal mapping does not exist and one selects quasi-conformal mapping instead to get a dielectric medium. The best-known application of quasi-conformal mapping is the carpet cloak which can also be used for guiding a plasmonic wave over a bump^6–8^. In this device, due to conformal module mismatch, a conformal map cannot be found. While conformal mapping leads to zero anisotropy by definition, the anisotropy introduced by quasi-conformal transformation can be made very small^3,26,45^.

The designs based on (quasi-) conformal TO can suffer from several drawbacks that are also common among non-conformal TO. Since any alteration to the coordinate lines at the boundaries is translated into changes of refractive index, using (quasi-)conformal TO leads in some cases to index mismatch between the device and the surrounding, meaning that power loss becomes unavoidable. This effect has been observed in devices where the boundary that interacts with the incoming or outgoing wave gets flattened, compressed or expanded to achieve directivity enhancement^14–17^ or beam compression^20,21^. In addition, employing TO often leads to less-than-unity index in a region of space where expansion occurs. There are three common approaches to deal with this problem. The first solution is to use resonant metamaterials, which, however, limits the bandwidth. The second one is to cut the region of space where the index is low or use unity index instead. This can affect the device performance, especially if that region interacts greatly with the wave^15^. The third solution is to multiply the whole index profile by a constant such that the index raises above unity. Being the most reasonable approach, matching of the device to the surrounding medium can be lost. When using quasi-conformal TO, even though the amount of anisotropy is minimized by this method, the minimum anisotropy value depends on how abruptly the mapping changes between different regions. For practical purposes, one neglects the small amount of anisotropy to have an isotropic device. It has been shown that this approximation can degrade the performance of the device. For instance, it can create an unwanted lateral shift to the light rays and render the device detectable in the case of carpet cloak^46^.

Here, we aim to propose a design method to remedy the defects that two devices, namely waveguide coupler, and plasmonic bump cloak, suffer from. A waveguide coupler is a device that can couple modes between two waveguides with different cross-sections and filled with desired dielectric constants. If the coupler is designed purely by TO, it can only couple two waveguides with predefined dielectric constants, governed by the ratio of cross-sections^20^. This is problematic if we need to have the desired medium at coupler’s ends. The impedance mismatch issue for a waveguide coupler has been investigated and addressed thoroughly in the literature^22–24^. Also, the coupler index at the conjunction with the waveguides will not be uniform, which makes the matching even more difficult^21^. Additionally, the design leads to less-than-unity index regions in general, which is not favorable. Note that multiplying the index by a constant is not practical here, since it changes the index at the boundaries as well, causing reflections. An interesting solution to manipulate the refractive index called artificial boundary conformal mapping (ABCM) has been proposed for the design of a waveguide crossing^47^. This method employs boundary modification and optimization to achieve better refractive index at the edges and also enhances the matching and uniformity of the refractive index at the input and output ports. Despite being elegant, using this method will not entirely eliminate the restriction imposed inherently by TO, e.g., the ratio of refractive index on two sides of a coupler being predefined by the compression ratio.

Here, a conformal TO is employed to acquire the refractive index. This is done in such a way to have a uniform index at the input and output boundaries, which is essential for the next step. To override the predefined matched dielectric constants for the waveguides and mitigate the less-than-unity index, the optical path is rescaled to match the device to vacuum or other desired dielectrics. At the same time, the rescaling helps to lower the maximum index value. The second device—the plasmonic cloak—has so far suffered from both the presence of less-than-unity index regions, and the anisotropy neglecting defects, the latter being not so prominent. Rescaling the optical path in a similar way as in the case of the waveguide coupler, we propose a conformal design and resolve the less-than-unity index issue, making the device much more practical.

## Transformation Optics and Conformal Mapping

One of the key ingredients of our method is transformation optics. TO relates the electromagnetic fields and the material between two spaces, namely virtual space [with Cartesian coordinates (*u*, *v*, *w*)] and physical space [with Cartesian coordinates (*x*, *y*, *z*)]. Virtual space is homogeneous (such as vacuum), where the light rays follow straight lines. Physical space is the result of applying a given mapping to the virtual space. The physical space relative permittivity and permeability tensors *ε* and *μ* are calculated from the ones of virtual space using the well-known formula^1^:

1 $$\varepsilon =\mu =\frac{J{J}^{T}}{{\rm{\det }}\,(J)}.$$

where *J* = ∂(*x*, *y*, *z*)/∂(*u*, *v*, *w*) is the Jacobian matrix.

In a two-dimensional (2D) conformal scenario, the mapping between virtual and physical spaces is chosen such that *z* = *w*, and for each *w* the plane (*u*, *v*, *w*) is conformally mapped to the plane (*x*, *y*, *z* = *w*). This way, the transformation is effectively performed only in two dimensions where a plethora of conformal mappings exists based on complex analytic functions. If we define the complex variables *W* = *u* + i*v* and *Z* = *x* + i*y*, we can express the mapping from virtual to physical space in terms of an analytic function *Z*(*W*) with the inverse function *W*(*Z*). Equation (1) then yields for this case the tensors *ε* = *μ* = diag (1, 1, |d*W*/d*Z*|^2^). Importantly, when one restricts the waves to TE polarization (electric field parallel to *z*-axis), the required optical mapping can alternatively be achieved by a purely dielectric material (*μ* = 1) with refractive index^14,29^

2 $$n(x,y)=\sqrt{\varepsilon (x,y)}=|\frac{{\rm{d}}W(Z)}{{\rm{d}}Z}|.$$

This is because the TE wave is only affected by the tensor components *μ*_*xx*_, *μ*_*yy*_ and *ε*_*zz*_, so changing the other components does not affect the wave in any way. One can, therefore, work with a purely dielectric material in the 2D conformal TO, which we will do in the following. Moreover, in many situations this is possible even for TM waves, in particular, if the refractive index does not change significantly on the scale of a wavelength. In such situations the wave has time to adjust to the slowly (adiabatically) changing medium, and polarization effects are not very important. This way, a purely dielectric material of Eq. (2) often works very well for both polarizations.

It is worth mentioning that for 2D problems, one can employ conformal mapping easier compared to 3D cases. Based on Riemann’s mapping theorem, simply-connected 2D regions are conformally equivalent to each other. However, finding the corresponding conformal map can be difficult. Liouville’s theorem, on the other hand, implies that as the dimension of the problem increases to more than 2, the number of existing conformal maps becomes substantially limited. Conformal 3D maps are limited to scaling, translation, and spherical inversion, which have very few applications for designing electromagnetic elements. The bright side is that for many 3D devices, the constraints of the problem allow the device to have symmetry around its optical axis, and hence conformal mapping can still be beneficial. For such cases, one carries out the design for 2D and revolves the material around the optical axis to create the 3D device. Devices that act as couplers and beam expanders and compressors or devices that generate directive beams in 3D are good examples of such scenarios.

## Waveguide Coupler Design Method

In the following, we present the method of designing the waveguide coupler. The first step is to establish virtual and physical spaces, and a conformal mapping between them; in the second step, we then perform rescaling of the optical path to modify the refractive index profile. To be able to present our method clearly, we have chosen particular coupler shape as well as values of different parameters; however, the method is general and flexible and the parameters and dimensions can be chosen almost arbitrarily. For this purpose, we also introduce the length parameter *a* that determines the size of the device.

**Figure 1:**
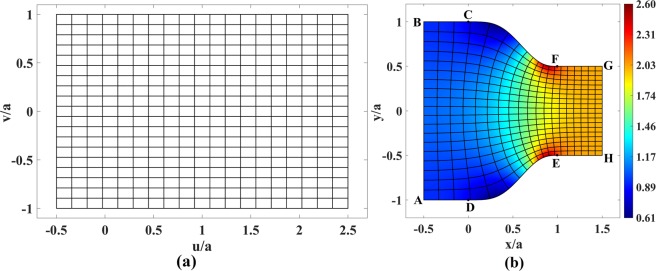
Schematics of the virtual and physical spaces for a coupler. (**a**) Rectangular virtual space with lines of constant *u* and *v*. (**b**) Physical space based on conformal mapping together with the corresponding mapped lines of constant *u* and *v* of Fig. 1(a). The refractive index is also shown in this figure.

Our virtual space is a rectangle filled with vacuum shown in Fig. 1(a). Physical space consists of three parts: rectangles ABCD and EFGH, and the shape CDEF [see Fig. 1(b)]. The top boundary of CDEF is defined by the following smooth function in the interval of 0 ≤ *x* ≤ *a*:

3 $$t(x)=[(1-C)(4{\cos }^{3}(\pi x/2a)-3{\cos }^{4}(\pi x/2a))+C]a,$$

where *C* denotes the amount of compression; in our example, *C* = 0.5. The function *t*(*x*) has zero first and second derivatives at *x* = 0 and *x* = *a* and also satisfies *t*(0) = *a* and *t*(*a*) = *aC*. The first and second derivatives are set to zero to let the curves CF and DE adapt to their straight-line continuations as smoothly as possible.

To find the conformal mapping between virtual and physical spaces, we use the fact that a transformation between two quadrilaterals can be conformal only if they share the same conformal module^48^. The conformal module (denoted here as *M*) for a rectangle is simply its aspect ratio. For the quadrilateral shape of physical space, *M* has to be calculated numerically by solving the following boundary-value problem for a function *ϕ*(*x*, *y*) in physical space:

4 $$\begin{array}{l}{\nabla }^{2}\phi =0\\ \phi =0\,{\rm{on}}\,HA\\ \phi =1\,{\rm{on}}\,BG\\ \partial \phi /\partial N=0\,{\rm{on}}\,AB\,{\rm{and}}\,GH,\end{array}$$

where *N* is the normal vector to the boundary. The conformal module *M* is then calculated from the following integral over physical space^48^:

5 $$M=\int {\int }_{Z}{|\nabla \phi |}^{2}dx\,dy=\int {\int }_{Z}[{(\frac{\partial \phi }{\partial x})}^{2}+{(\frac{\partial \phi }{\partial y})}^{2}]dx\,dy.$$

The calculation can be carried out using MATLAB or COMSOL Multiphysics PDE solvers. For our example of physical space in Fig. 1(b), the conformal module is *M* = 1.5. This then sets the aspect ratio of the rectangular virtual space, and has already been taken into account when setting the dimensions of Fig. 1(a).

The conformal mapping between the physical and virtual spaces, in this case, cannot be presented by a closed-form formula and should be calculated numerically by solving Laplace equation in physical space for variables *u* and *v*^3,15,25–28^. The Laplace equation and the Dirichlet and Neumann boundary conditions for this case are defined by the following equations:

6 $$\left \{\begin{array}{c}{{\rm{\nabla }}}^{2}u=0\\u=-a/2{\ {\rm on} \ }AB\\ u=5a/2{\ {\rm on} \ }GH\\{\rm{\partial }}u/{\rm{\partial }}N=0{\, on}BG{and\, }HA\end{array},\right.\left\{\begin{array}{c}{{\rm{\nabla }}}^{2}v=0\\ v=-a{\ {\rm on} \ }HA\\v=a{\ {\rm on} \ }BG\\{\rm{\partial }}v/{\rm{\partial }}N=0{\ {\rm on} \ }AB{and\, }GH\end{array}.\right.$$

The Neumann boundary condition ensures that the mapped virtual space lines maintain the orthogonality to the corresponding boundaries.

The refractive index in physical space is then calculated by Eq. (2) and is shown in Fig. 1. As expected, the index reaches values below unity in regions where the space is expanded and more than unity where space is compressed. The minimum and maximum values can be altered to possess more moderate values by modifying design parameters like decreasing the amount of compression, or making the CDEF shape wider. As it can be seen from Fig. 1, the device is intrinsically matched to the refractive index of 1 and 1/*C* = 2 at the left and right boundaries, respectively. This holds true for all kinds of transformation-optical coupler designs^20^.

### Rescaling the optical path

Having established the conformal mapping between virtual and physical spaces, we now proceed to the second step to solve two problems at the same time: we will match the index on the right-hand side to unity, as well as remedy the less-than-unity index issue.

Consider a plane wave propagating in virtual space from left to right. The vertical and horizontal lines in virtual space of Fig. 1(a) then represent the phase-fronts and the ray trajectories, respectively. By the essence of TO, this is therefore also true for their corresponding mapped lines in physical space. We see that the phase-front lines in physical space are at the same time contours of the function *u*(*x*, *y*) that corresponds to coordinate *u* of virtual space. And just as this coordinate *u* measures the optical path length along the ray in virtual space, the corresponding *u*(*x*, *y*) measures the optical path length along the ray in physical space. The refractive index then corresponds to the rate at which the optical path changes along the ray:

7 $$n(x,y)=|\nabla u|,$$

which is in accord with Eq. (2), taking into account the Cauchy-Riemann conditions.

Now comes the key step of our construction. We define an increasing function *S*(*u*) (we will call it *scaling function*) and imagine that it is *S* rather than *u* that measures the optical path length along the ray in physical space. The contours of *S* are the same as those of *u*, so the phase-fronts are not modified by this change, and therefore rays are not modified either. However, since the rate at which the optical path is growing along the ray has changed, so must do the refractive index. The resulting new refractive index *n*′ will then be

8 $$n^{\prime} (x,y)=|\nabla S|=|\frac{dS}{du}|n(x,y),$$

where we have used Eq. (7). This construction gives great design freedom: by choosing a suitable function *S*(*u*), we can modify the refractive index such that it matches vacuum at the boundaries, and also shift its minimum value above unity to avoid superluminal propagation.

**Figure 2:**
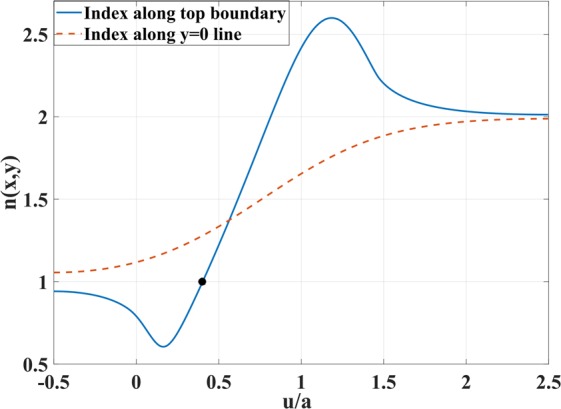
Refractive index in Fig. 1(b) versus *u*/*a* along the top boundary and *y* = 0 lines.

To demonstrate this, we take as reference the upper boundary of the coupler which contains the minimum and maximum values of the index *n*. Figure 2 shows the refractive index *n* as a function of *u*/*a* at the top boundary of physical space and on the *y* = 0 line. It is desirable to have unity refractive index on both the left and right sides so that the coupler is matched to the vacuum. We separate the index curve of the top boundary into two parts according to the sign of *n* − 1, and label the points *u*_0_ = −*a*/2 (left boundary), ${u}_{1}\simeq 2a/5$ (the point where *n* = 1) and *u*_2_ = 5*a*/2 (right boundary). The index *n* is less than one on the segment *u*_0_*u*_1_ (the point *u*_1_ is marked in Fig. 2 with a black dot). For *u* ∈ [*u*_0_, *u*_1_], we employ the following scaling function that makes the index equal to unity on the upper boundary:

9 $$S(u)={\int }_{-a/2}^{u}\,\frac{1}{n(u)}{\rm{d}}u.$$

**Figure 3:**
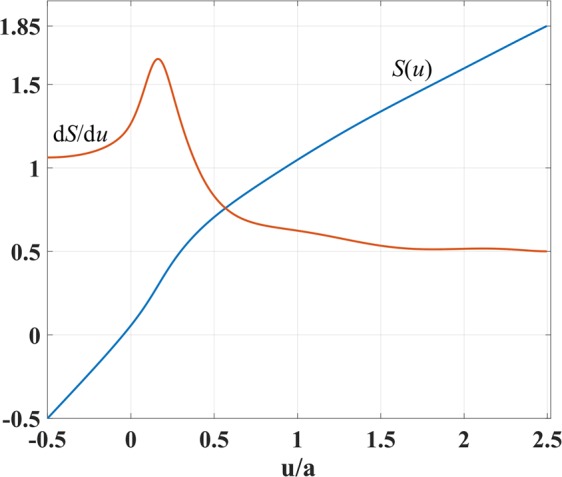
Scaling function *S*(*u*) and its first derivative d*S*/d*u* versus *u*/*a*.

As for the interval *u* ∈ [*u*_1_, *u*_2_], we cannot employ the same condition because shifting the index to unity in the concave region near points E, F in Fig. 1 would lead to the index dropping below unity in the region between points E and F. So our goal for the interval *u* ∈ [*u*_1_, *u*_2_] is to minimize the refractive index while keeping it above unity. To do this, we have expressed *S*(*u*) as a polynomial series of degree six. The scaling function value and the first and second derivatives are chosen to be continuous at point *u*_1_ for a smooth change of refractive index. Also, the first derivative value at *u*_2_ equals ${\rm{d}}S/{\rm{d}}u{|}_{{u}_{2}}=0.5$ to make the index *n*′ unity there, and the second derivative is taken to be zero for smoothness. We apply an optimization for the derivation of polynomial coefficients based on the the above constraints. The index profile *n*(*x*, 0) along the line *y* = 0 is considered during the optimization to prevent the index *n*′(*x*, 0) from dropping below unity inside the coupler domain. Figure 3 represents the scaling function *S*(*u*) and its first derivative d*S*/d*u*.

**Figure 4:**
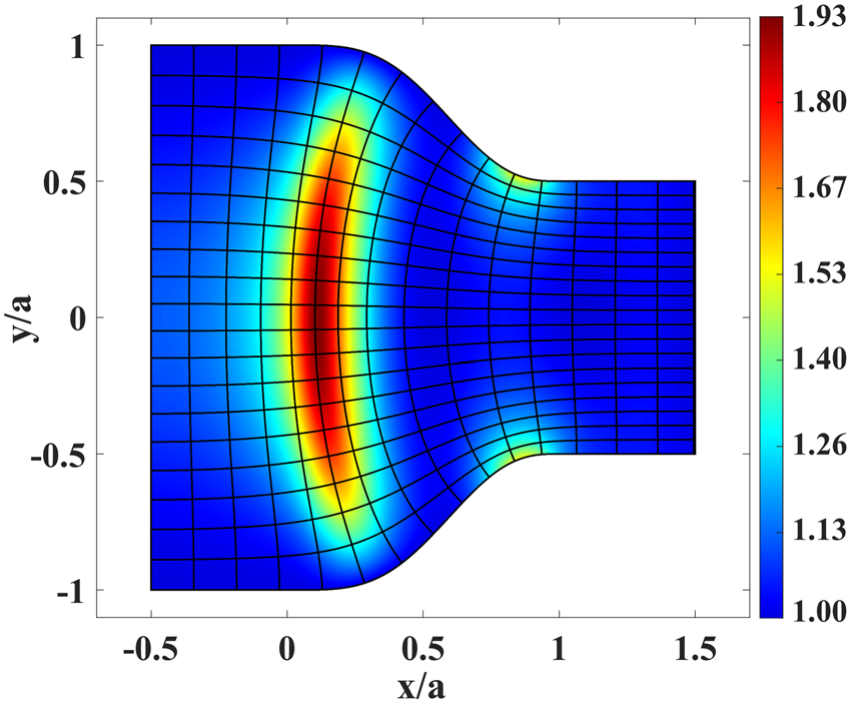
Refractive index of the coupler in Fig. 1(b) after scaling the phase-front contours, together with the equidistant contours of *S*(*u*(*x*, *y*)).

Figure 4 presents the resulting refractive index *n*′(*x*, *y*) in physical space based on Eq. (8). It can be seen that the index does not drop below unity and reaches unity at the left and right coupler boundaries. Moreover, the maximum required refractive index is considerably reduced. Equidistant contours of the function *S* are depicted in Fig. 4 as well.

## Plasmonic Bump Cloak

**Figure 5:**
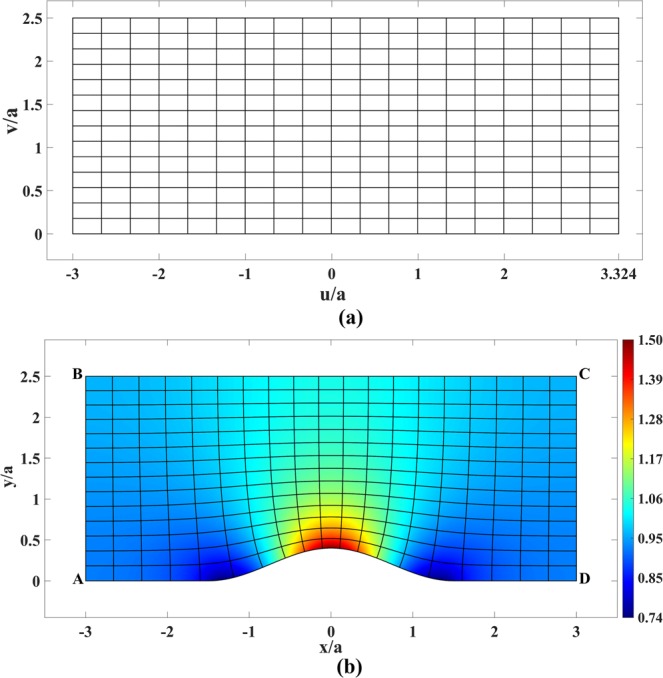
Virtual and physical spaces for a plasmonic bump cloak. (**a**) Rectangular virtual space with lines of constant *u* and *v*. (**b**) Physical space based on conformal mapping together with the corresponding mapped lines of constant *u* and *v* of (**a**). The refractive index is also shown in this figure.

Now we turn our attention to the plasmonic bump cloak. For such a device, virtual space is a rectangle and the physical space is a rectangle with a missing bump at the bottom side, see Fig. 5. In our example, the dimensions of physical space are 6*a* × 5*a*/2, where again *a* sets the size of the cloak and the bump. The bump boundary is given by the function *y*(*x*) = *h*_0_cos^2^(*πx*/*al*) with the height *h*_0_ = 0.4*a* and length of *l* = 3*a*. The virtual space lines of constant *u* and *v* are illustrated together with their corresponding mapped lines in physical space. Compared to the previous articles in the literature^6–8^, the bump here is two times taller and 1.5 times wider relative to the cloak size.

Similar to the case of the coupler, we calculate the conformal module *M* for physical space. Having *M*, we can set the width of virtual space such that the aspect ratio of virtual space equals *M*. Using Eqs. (4 and 5), the value of *M* = 2.53 is found for physical space and the length of virtual space in Fig. 5(a) is chosen according to this value.

To find the conformal map between two spaces, the following sets of equations are solved:

10 $$\left \{\begin{array}{l}{\nabla}^{2}u=0\\ u=-3a{\ {\rm on} \ }AB\\ u=3.32a{\ {\rm on} \ }CD\\ {\partial}u/{\partial} N=0{\ {\rm on} \ }BC {\ {\rm and} \ } DA\end{array}, \right. \left \{\begin{array}{l}{\nabla}^{2}v=0\\ v=0{\ {\rm on} \ }DA\\ v=5a/2{\ {\rm on} \ }BC\\ {\partial}v/{\partial}N=0{\ {\rm on} \ }AB{ \ {\rm and} \ } CD\end{array}.\right.$$

**Figure 6:**
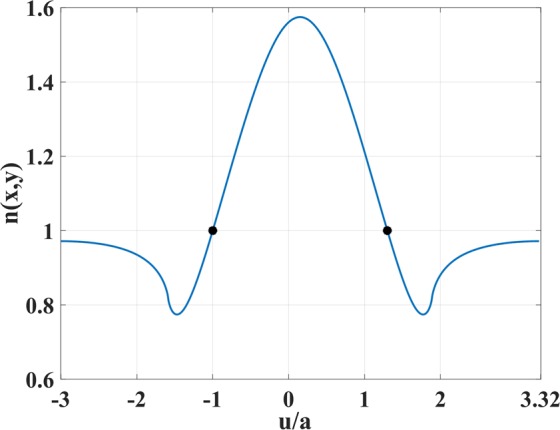
Refractive index in Fig. 5(b) versus *u*/*a* along the bottom boundary line.

**Figure 7:**
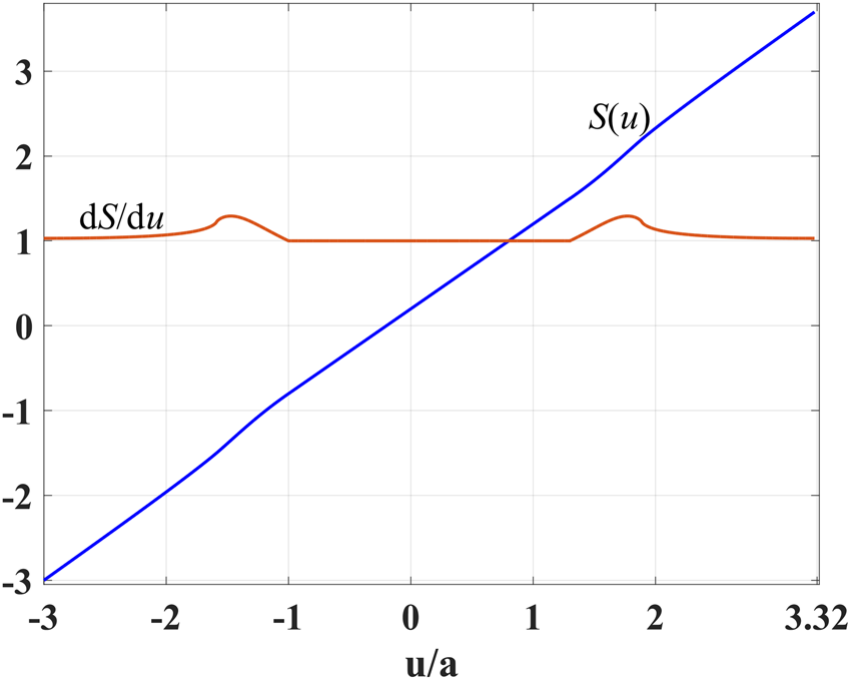
Scaling function *S*(*u*) and its first derivative d*S*/d*u* versus *u*/*a*.

**Figure 8:**
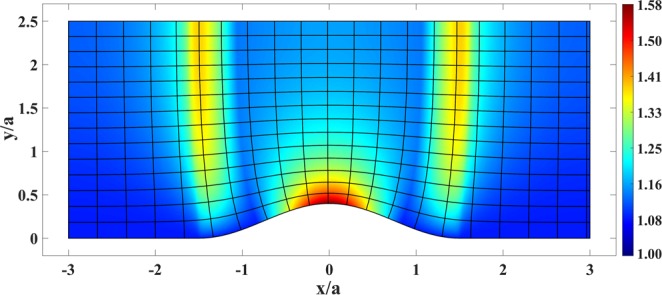
Refractive index of the plasmonic cloak in Fig. 5(b) after scaling the phase-front contours. The equidistant contours of *S*(*u*(*x*, *y*)) are plotted as well.

The refractive index for this design is shown in Fig. 5(b) and it covers the range of 0.74 ≤ *n* ≤ 1.5. The index along the bottom boundary is sketched in Fig. 6 that also marks the points ${u}_{1}\simeq a$ and ${u}_{2}\simeq 1.3a$ where the index reaches unity. The initial and final values of *u* are labeled as *u*_0_ = −3*a* and *u*_3_ = 3.32*a*, respectively. Again, we apply the scaling to remove the index values below unity, working separately in intervals *u* ∈ [*u*_0_, *u*_1_] and *u* ∈ [*u*_2_, *u*_3_] in a similar way as for the coupler. For the interval *u* ∈ [*u*_1_, *u*_2_] we applied no scaling since it was not necessary. Moreover, lowering the index in this region would lead to less-than-unity index *n*′ on the top boundary of physical space, which is not desirable. Figure 7 represents the resulting scaling function *S*(*u*) and its first derivative d*S*/d*u*. Using Eq. (8), the refractive index *n*′(*x*, *y*) of the device is calculated, and plotted in Fig. 8. The equidistant contours of the function *S* are depicted in Fig. 8 as well.

## Simulation Results

**Figure 9:**
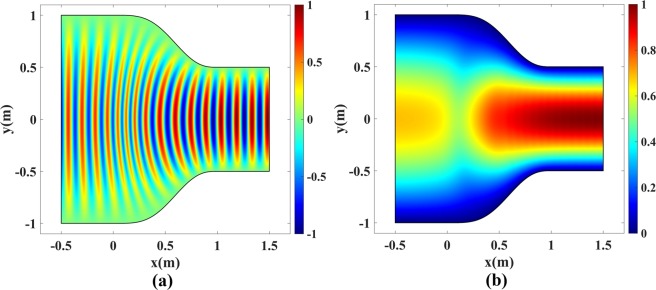
Normalized electric field of the TE_1_ mode for the waveguide coupler in Fig. 4. (**a**) Real part and (**b**) Magnitude of *E*_*z*_.

**Figure 10:**
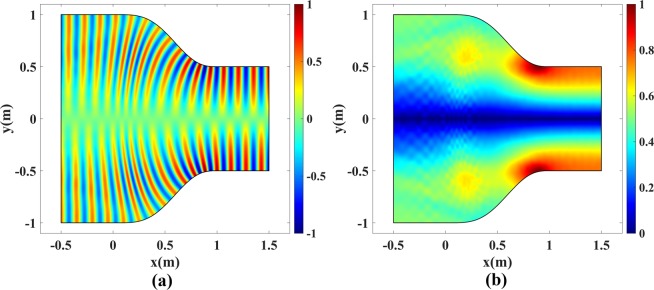
Normalized magnetic field of the TM_1_ mode for the waveguide coupler in Fig. 4. (**a**) Real part and (**b**) Magnitude of *H*_*z*_.

We have tested the performance of the coupler and the plasmonic bump cloak designed in the previous sections by multiple numerical simulations in COMSOL. First, the functionality of the waveguide coupler in Fig. 4 is investigated for the value *a* = 1 m, and microwave operating frequency of 2 GHz. The device is excited with TE_1_ and TM_1_ modes, respectively, using a port on the right side. A perfect electrical conductor (PEC) is placed at the top and bottom boundaries to set the proper boundary conditions for TE and TM mode propagation, and the scattering boundary condition is used on the left boundary. The normalized real part and magnitude of electric fields for the TE_1_ mode are depicted in Fig. 9. The normalized real part and norm of magnetic fields for the TM_1_ mode are illustrated in Fig. 10. The reflection coefficients for both simulations are negligible and the modes are perfectly coupled from one side to the other. Note that if one uses the pure TO device in Fig. 1(b) with refractive index at the right side equal to 2, the reflection coefficient will approximately equal (2 − 1)/(2 + 1) = 1/3.

**Figure 11:**
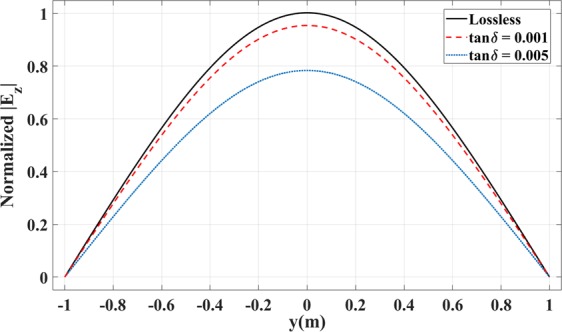
Normalized Magnitude of *E*_*z*_ on the output left boundary for the waveguide coupler in Fig. 4 for the lossless case (solid black line) and lossy cases with tan*δ* = 0.001 (red dashed line) and tan*δ* = 0.005 (blue dotted line).

To include the effect of loss, we have simulated the waveguide coupler in Fig. 4 for the case of TE polarization, and we have taken into account the loss tangent. Figure 11 illustrates the results for the normalized electric field component *E*_*z*_ for three cases with loss tangent values of tan*δ* = 0 (lossless), tan*δ* = 0.001, and tan*δ* = 0.005. As expected, loss introduces amplitude reduction to the structure. Note that the normalization is done with respect to the lossless case.

**Figure 12:**
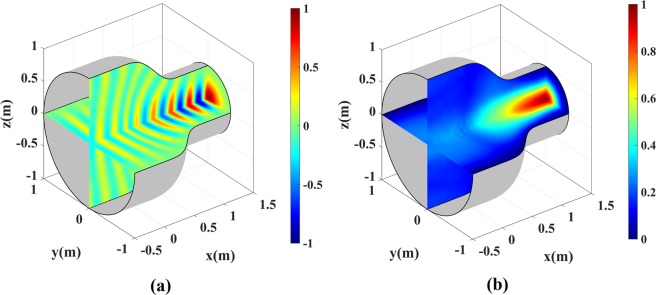
Normalized electric field for the 3D waveguide coupler. The Gaussian wave with *z* polarization is launched at the narrower side. (**a**) Real part and (**b**) Magnitude of *E*_*z*_.

The simulation of a rotationally-symmetric version of the coupler is carried out next. The medium is revolved around its optical axis and the PEC boundaries are removed. A Gaussian wave with polarization along *z*-axis in then launched at the narrower side of the coupler. The reduced simulation frequency of 1 GHz is chosen to handle the numerical costs of the simulation. The real part and magnitude of the electric field for this case are represented in Fig. 12.

**Figure 13:**
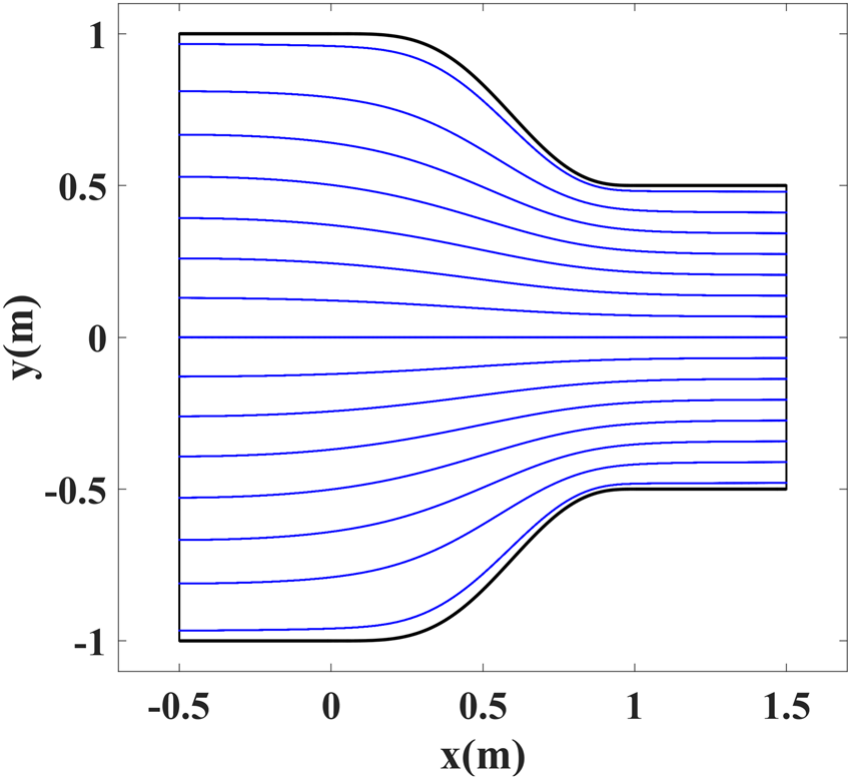
Ray trajectories inside the coupler of Fig. 4.

The last simulation for the case of waveguide coupler includes the ray-tracing simulation. This simulation verifies the high-frequency behavior of the device and is based on geometrical optics approximation. As mentioned before, the ray trajectories before the scaling are the mapped lines of constant *v* in Fig. 1(b). The ray trajectories after the scaling are shown in Fig. 13 where fifteen rays are launched from the right boundary of the coupler with equal spacing. This figure demonstrates that the scaling has no effects on the ray trajectories while it alters the refractive index to our desired fashion.

The plasmonic bump cloak is the next simulation case. Parameter *a* = 1 *μ*m is selected to set the device size. An out-of-plane plasmonic magnetic field is launched from the left boundary of the device in Fig. 8. The simulation is carried out for the operating wavelength of 633 nm. The bottom conductor is a gold slab with a thickness of 100 nm^49^. The TM plasmonic wave is excited at the left boundary^50^.

**Figure 14:**
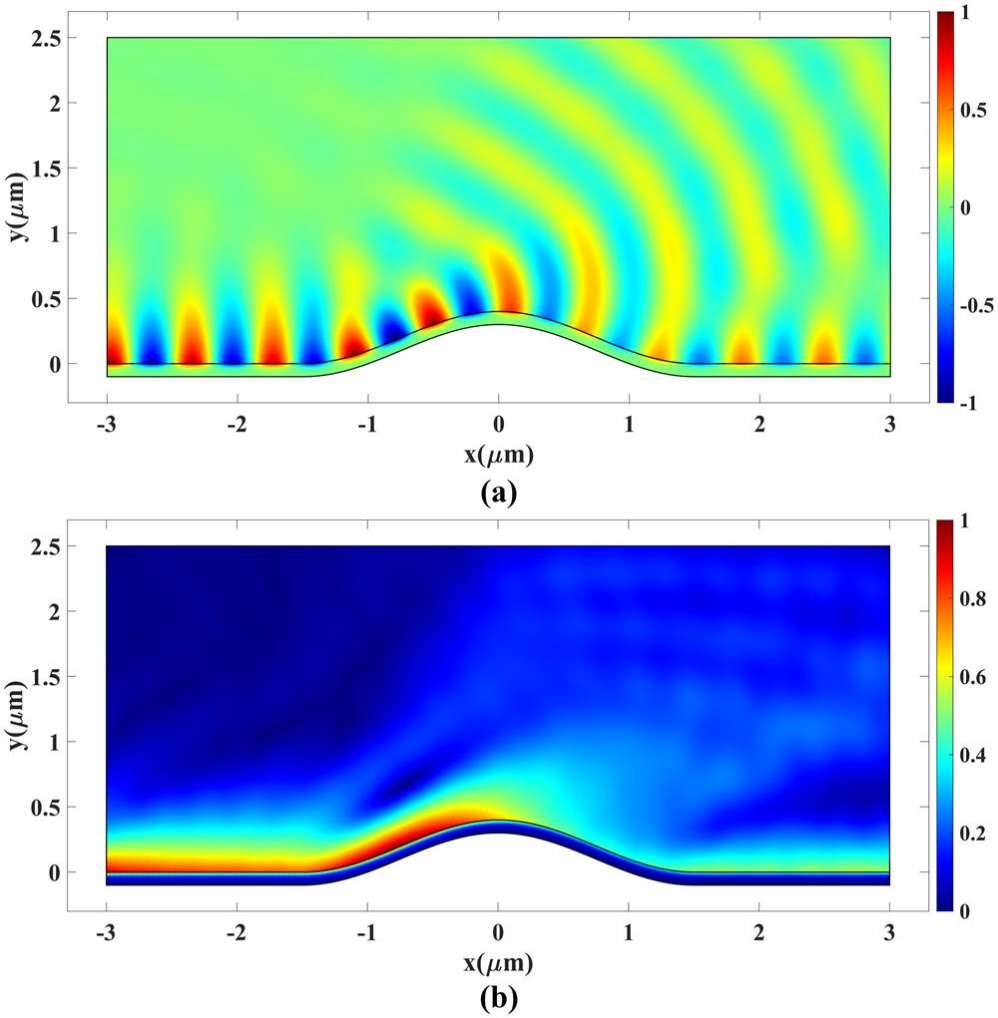
Plasmonic wave propagation over a bare bump. (**a**) Real part and (**b**) Magnitude of *H*_*z*_.

Figure 14 illustrates the plasmonic wave propagation over a bare bump. Numerical calculation of the power reveals that more than 53% of the plasmonic wave power is scattered due to the presence of the bump.

**Figure 15:**
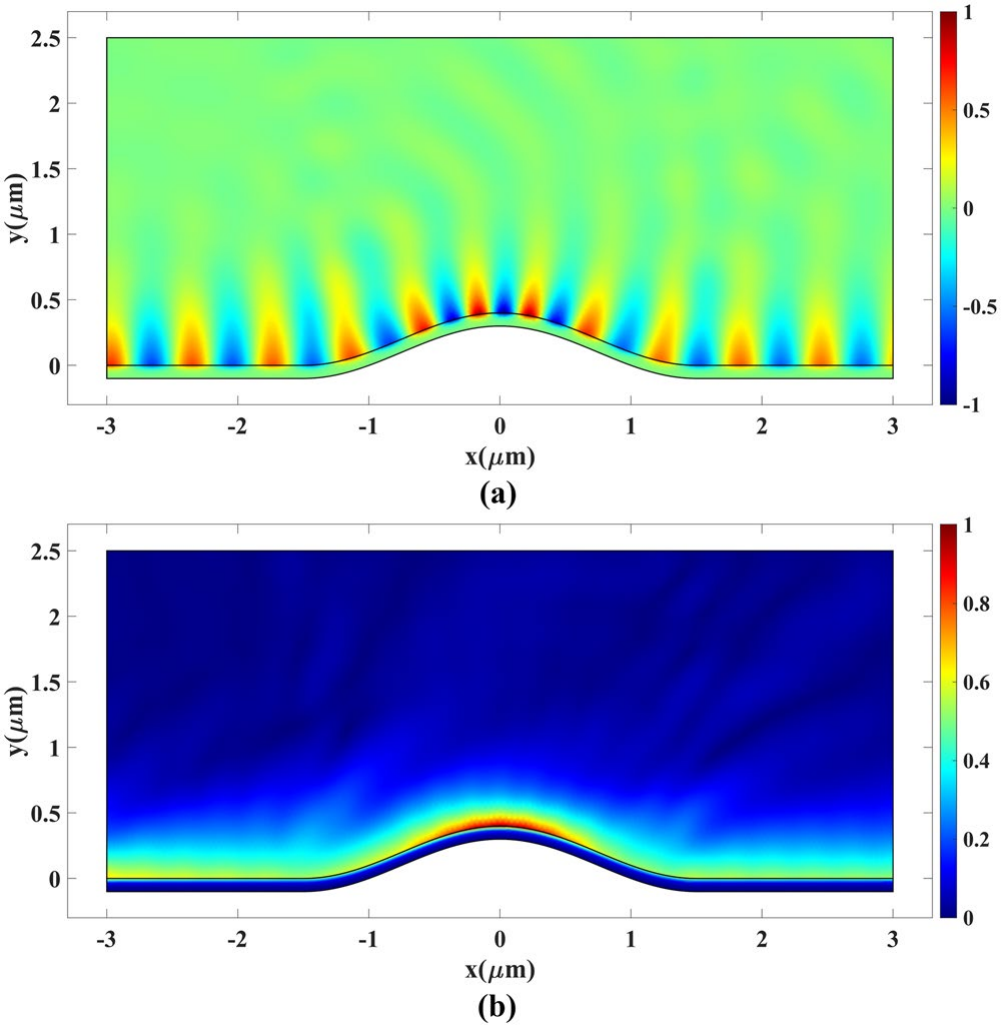
Plasmonic wave propagation over a bump covered with the cloak of Fig. 8. (**a**) Real part and (**b**) Magnitude of *H*_*z*_.

Next, the rescaled plasmonic bump cloak in Fig. 8 is used to conceal the bump from the incoming plasmonic wave. Figure 15 illustrates the results for this case. It can be seen that the scattering due to the bump is negligible. It is calculated numerically that less than 4% of the input power is scattered.

## Conclusion

In conclusion, we have proposed a method of designing optical devices that have the same functionality as previously designed devices, but with much more moderate ranges of refractive index. The key ingredients of the method are the conformal mapping between virtual and physical spaces, and rescaling the optical path along the rays. The method enables to eliminate superluminal regions of space as well as lower the refractive index in other regions to achievable values. It provides a lot of freedom that can be employed to optimize the refractive index profile to match the desired purpose. Numerical simulations confirm our theoretical results very well and show that the devices proposed work very well even for different polarizations.

Having said this, we must note that the presented design method is based on rescaling the index for one particular bunch of rays. These rays will be unaffected by the rescaling, but other rays, in principle, will be affected. Therefore our method is useful in situations when light is propagating only in a certain direction; it cannot be used to modify e.g. imaging systems that should focus light coming from different directions. However, we think that there are still many practical situations when the method will be useful—a particular example is a coupler that enables one to connect smoothly waveguides of different sizes without the necessity of high refractive index regions. We hope that our method will find other uses too, for example in designing horn antennas.
